# The titanium elastic nail serves as an alternative treatment for adult proximal radial shaft fractures: a cohort study

**DOI:** 10.1186/s13018-017-0704-y

**Published:** 2018-01-15

**Authors:** Ying-Cheng Huang, Jenn-Huei Renn, Yih-Wen Tarng

**Affiliations:** 1 Department of Orthopaedics, Kaohsiung Veterans General Hospital, No. 386, Ta-Chung 1st Rd, Kaohsiung, 81346 Taiwan, Republic of China; 2 National Yang-Ming University, Taipei, Taiwan, Republic of China; 3 National Defense Medical Center, Taipei, Taiwan, Republic of China

**Keywords:** Proximal radial fracture, Titanium elastic nail, TEN, Radial intramedullary nail, Radial interlocking nail

## Abstract

**Background:**

To investigate whether closed reduction and internal fixation (CRIF) with titanium elastic nails (TENs) is a viable alternative treatment in proximal radial fractures.

**Methods:**

In Kaohsiung Veterans General Hospital, from November 2013 to April 2015, five adult male patients with forearm injuries (average age 43 years; range 35–64 years) were treated for proximal radial shaft fractures. CRIF with TENs for radial shaft fractures was performed in these five patients. Radiographs; range of motions; visual analog scale (VAS); quick disabilities of the arm, shoulder, and hand (Quick DASH) questionnaire; and time to union were evaluated in our study.

**Results:**

Mean follow-up period was 30 months (range 28–36 months). Average time of radius union was 7.3 months (range 6–10 months). Functional outcomes 1 year after operation revealed an average Quick DASH score of 7.92 (range 4.5–25), an average VAS of 1.5 (range 1–3), and average forearm supination and pronation of 69.2° (range 45°–75°) and 82.5° (range 80°–85°). No major complication was noted.

**Conclusions:**

CRIF with TEN for adult proximal radial fractures is a method to avoid extensive exposure or nerve injury during ORIF, especially in multiple trauma patients who require short operative time, uremia patients with ipsilateral forearm AV shunt, severe soft tissue swelling due to direct muscle contusion or strong muscularity before surgery, extensive radial fracture, and those in pursuit of cosmetic outcomes.

## Background

Diaphyseal forearm fractures are considered intraarticular fractures. Anatomic reduction with plates and screw fixation serves as the gold standard as it is important to restore rotational stability and preserve double bone length [1, 2]. However, extensive surgical exposure and periosteal stripping during open reduction surgery may increase the risks of neurovascular injuries, soft tissue injuries, intraoperative fractures, muscle swelling, and even postoperative compartment syndrome [3–5]. To avoid iatrogenic injuries, non-locked intramedullary nailing treatment for forearm fractures has been previously reported [5].

It is worth noting that the precontoured elastic stable intramedullary nail (such as titanium elastic nail (TEN) fixation for radial shaft fractures) is widely used in children as it is safer and more efficient compared with plating [6]. This technique preserves the periosteum, allowing bone healing within a closed and intact biological environment [7, 8]. By contrast, adult bone healing properties are diminished compared with that of children. Osteoblasts in the inner cellular layer of the child’s thick periosteum become thinner with age, and the bone healing process is also prolonged with aging.

The biomechanical principal of the TEN is based on the symmetrical bracing action of elastic nails inserted into the metaphysis, which bears against the inner bone at three points [9]. This method has the benefits of early immediate stability to the involved bone segment, which permits early mobilization and returns to the normal activities of the patients, with very low complication rate [10]. TENs lack axial and rotational stability but they are relatively stable with secondary bone healing. There are few reports evaluating the use of elastic stable intramedullary nails in adult proximal radial fractures [11, 12]. The proximal radius is surrounded by abundant forearm muscle, especially the supinator and pronator teres, and the posterior interosseous nerve (PIN) also crosses the proximal radius. The intramedullary nail method has advantages such as closed application, less soft tissue injury, avoidance of nerve injury, and cosmetic benefits. In particular, the application of the TEN for adult proximal radial shaft fractures has not been extensively investigated.

The underlying hypothesis of this study was that limited surgical dissection to treat adult proximal radial shaft fractures can avoid neuromuscular injury, reduce blood loss, enhance fracture healing, and yield better cosmetic results compared with the standard procedure of open reduction with plate and screw fixation. Toward this end, this study evaluated the functional outcomes and efficiency of TENs in the surgical treatment of adult proximal radial shaft fractures.

## Methods

The methodology of our study is a retrospective cohort study. The institutional review board of Kaohsiung General Veterans Hospital approved this retrospective study and informed consents were taken from all the patients. This study assessed patients with proximal radial shaft fractures who underwent fixation with TENs (DePuy Synthes, Johnson & Johnson Family of Companies, MA, USA) from November 2013 to April 2015. In total, five patients (six radial fractures) were included and anteroposterior (AP) and lateral forearm radiographs were obtained on first admission following trauma. All fracture patterns were recorded according to the Arbeitsgemeinschaft für Osteosynthesefragen/Orthopaedic Trauma Association (AO/OTA) fracture classification system.

All distal radial ulnar joint (DRUJ) stability was evaluated with radiographs and physical examinations before surgery. Postoperative DRUJ stability and neurological assessment were evaluated, and range of motion at the wrist, elbow, and forearm supination and pronation were recorded. Numbness or wrist drop indicated iatrogenic nerve injury. The details of evaluation of range of motion (ROM) of the forearm with the elbow flexed at 90° were measured by a goniometer [13].

Primary outcome measurement is the range of motion of forearm supination and pronation, and the secondary outcome measurements include the time to achieve bone union and the functional questionnaire to evaluate the function of diseased limb at 12 months postoperatively.

**Fig. 1:**
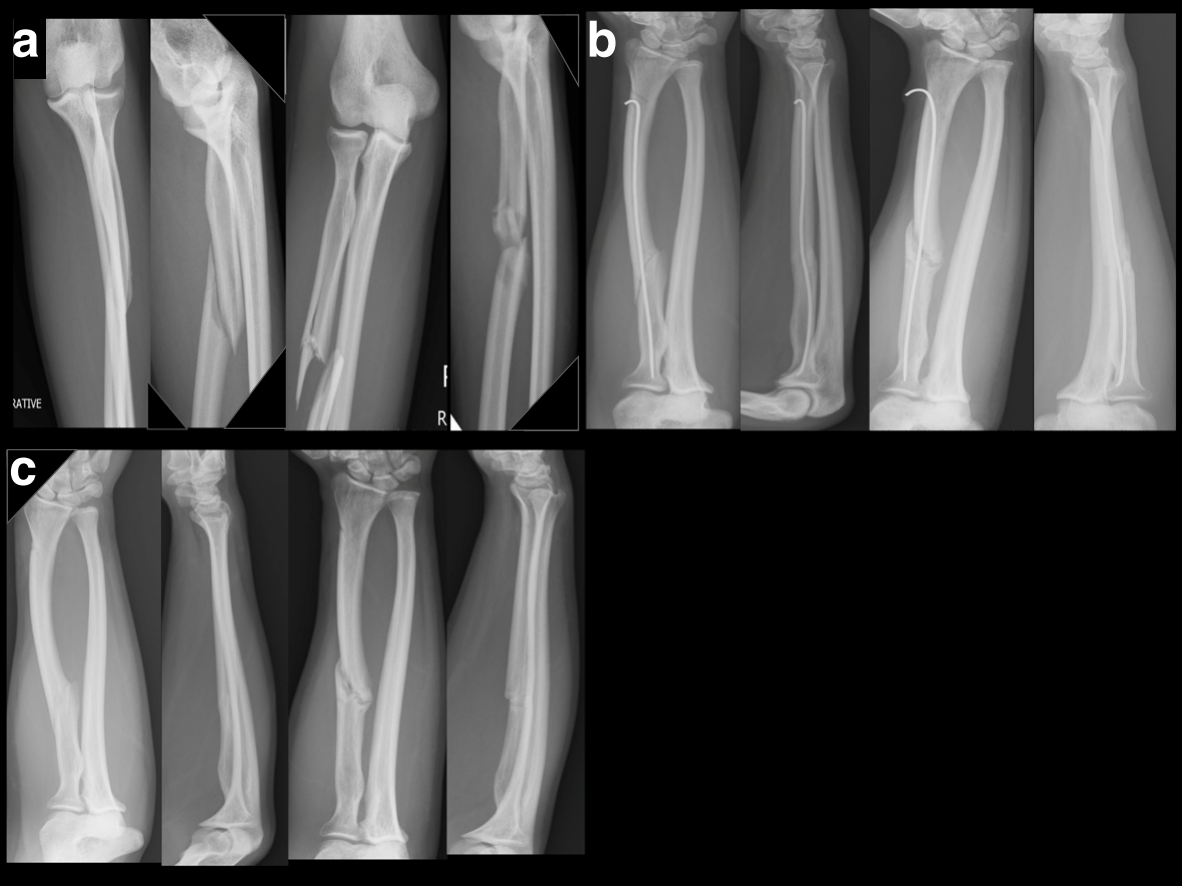
A 30-year-old male was involved in a traffic accident and sustained bilateral proximal radial shaft fractures. **a** Preoperative anteroposterior radiographs show bilateral proximal radial oblique diaphyseal fractures. **b** Six months postoperative views show TENs placed to stabilize the radial shaft fractures. Bridging callus is noted around the fractures sites. **c** Postoperative follow-up radiographs reveal bone union of bilateral proximal radial shaft fractures. Removal of the implants 8 months postoperation did not contribute to refractures

**Fig. 2:**
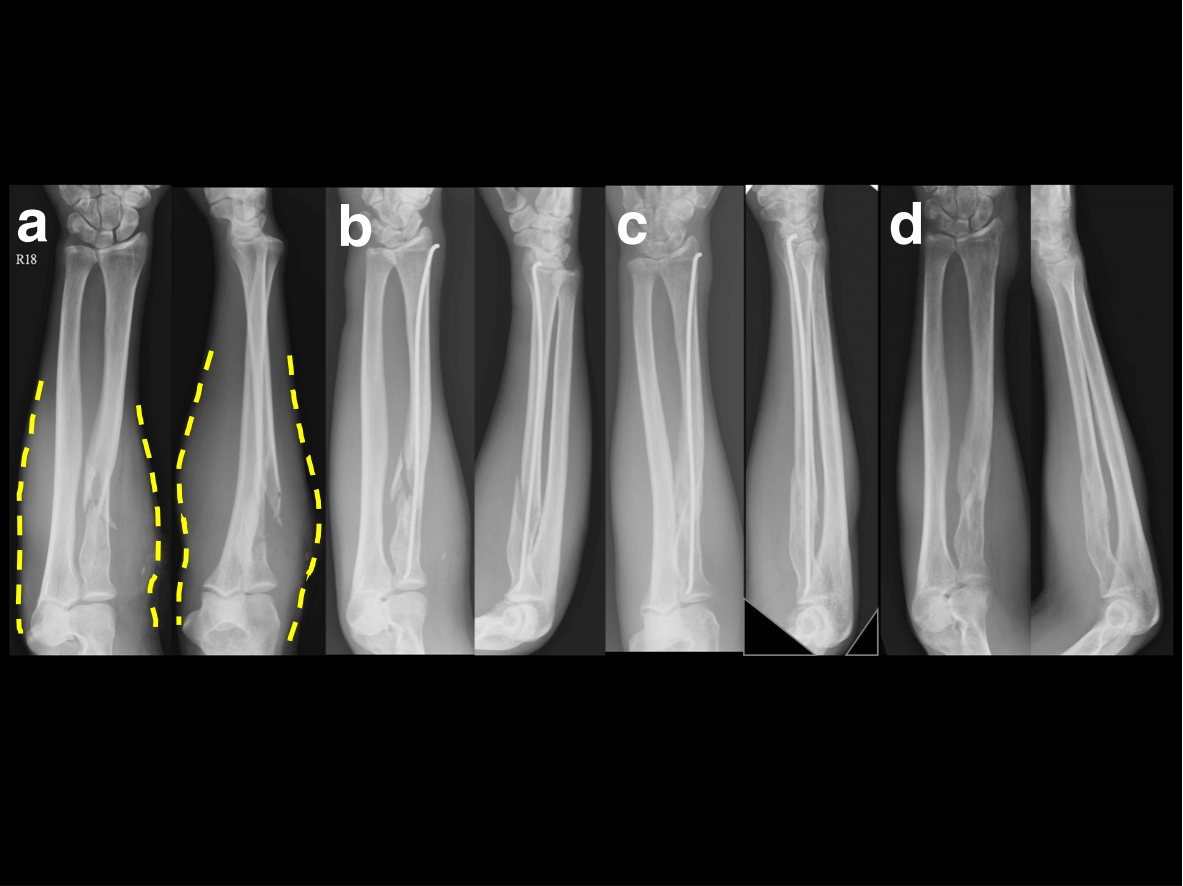
A 43-year-old male fell from his motorcycle with direct contusion of the right forearm and was diagnosed with right proximal radial comminuted fracture. **a** The dotted lines illustrate the severe soft tissue swelling over the proximal forearm. **b** Closed reduction is performed with TEN fixation. **c** Four months postoperative views show an acceptable radial arch with callus formation. **d** Eight months postoperative views show radiographic evidence of bone union

**Fig. 3:**
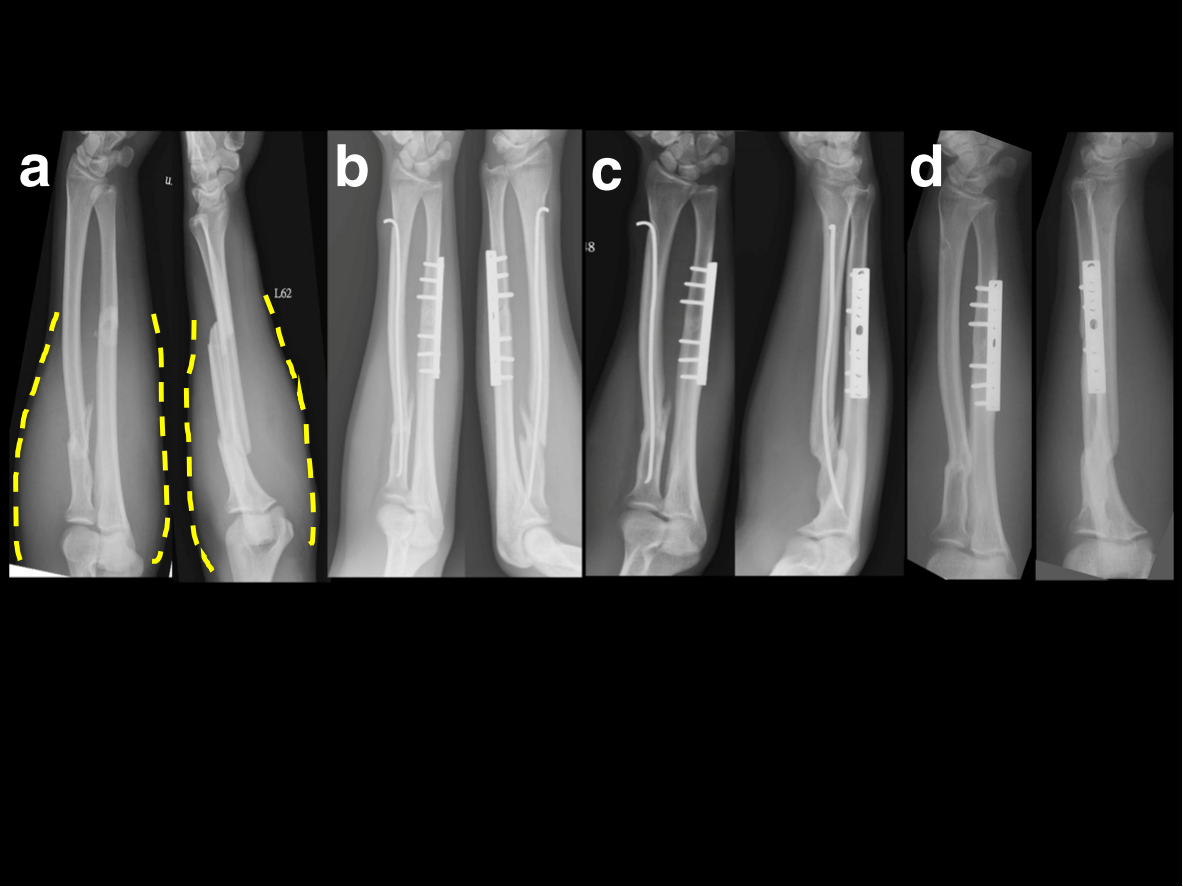
A 35-year-old male sustained a left radio-ulnar shaft fracture from a traffic accident. Preoperative radiographs of the forearm show a proximal radial transverse diaphyseal fracture and an ulnar shaft diaphyseal oblique fracture. **a** The dotted lines illustrate the severe soft tissue swelling. **b** Fractures are treated with open reduction and plate fixation for ulnar fracture and closed reduction and TEN fixation for radial fracture. **c** Four months postoperative views show malposition of radial arch with callus formation over the fracture site. **d** Eight months postoperative views shows radiographic bone union

In this study, one patient had bilateral extensive proximal-third radial shaft fractures (Fig. 1), one patient had only a right proximal-third radial shaft comminuted fracture (Fig. 2), and three patients had left proximal-third radial and ulnar shaft fractures (Fig. 3). All patients were male, and the average age was 43 years (range 30–64 years). Etiologically, all fractures were sustained during traffic accidents while riding a motorcycle.

### Surgical technique

After induction of appropriate anesthesia, the patient was placed in the supine position and a tourniquet was placed at the upper arm for the reduction of blood loss. The arm was placed on a radiolucent arm table or suspended vertically in traction. Surgical intervention began first with open reduction and internal fixation (ORIF) with plate fixation for ulnar shaft fractures to preserve forearm length if there were combined radio-ulnar fractures, and then radial fractures were treated via closed reduction and internal fixation (CRIF) with TEN fixation under the aid of an image intensifier (OEC Fluorostar 7900 Digital Mobile C-arm, GE Healthcare, UK). The diameters of the nail are about two thirds of the medullary isthmus of radius. In our study, a nail diameter of 2.5 mm is mostly used, whereas 3.0 mm nail is also used. The TENs were pre-bent into a “C” shape with the nail tip pointing toward the concave side of the bowed nail. A 1.5-cm skin incision was made below the radial styloid. The entry point was created proximal to the first dorsal component to avoid abductor pollicis longus and extensor pollicis brevis injuries. Penetrating near the cortex with the drill bit and using a rotating awl, the awl was then slowly lowered to an angle of 45° relative to the shaft axis and was advanced at this angle until it reached the medullary canal. An adequate length of TEN outside the skin was then temporarily fixed into a Synthes Universal Chuck with T-handle (DePuy Synthes, Johnson & Johnson Family of Companies, MA, USA). Using oscillating hand movements, the unreamed TEN was gently advanced manually in a retrograde manner until it reached the fracture site. The nail was introduced into the proximal fragment by indirect manipulation of the fragment under fluoroscopy. The nail was carefully advanced manually throughout the entire implantation procedure to avoid penetrating the cortex, especially in osteoporotic bone, until it rested at the radial tuberosity. When the nails were correctly positioned, the protruding nail ends were cut approximately 1 cm from the bone and then were pushed into their final positions using the impactor, with 5 mm of the nail end left beyond the cortex. TEN ends were left longer and placed sufficiently far outside the tendon compartment to avoid constant friction and tendon rupture. All TENs were removed after bone union.

### Postoperative care and follow-up

Postoperative care included (1) immobilization for each patient with a long-arm splint/cast in a forearm supination position for 4 weeks (splint for the first week postoperatively due to the soft tissue swelling, after the soft tissue swelling improved, casting from the second to fourth weeks postoperatively); (2) active/passive motion of the elbow and forearm rotation rehabilitation after removal of the cast; and (3) TENs removal when bone union was confirmed.

All patients were followed up for at least 2 years postoperatively. Close follow-up at our outpatient department included radiographs of the forearm to evaluate fracture healing and the recording of functional outcomes using the visual analog scale (VAS) for pain, the Quick Disabilities of the Arm, Shoulder, and Hand (Quick DASH) questionnaire [14], and the maximum ROM at the forearm using a goniometer was measured with supination and pronation at 12 months postoperatively.

## Results

According to the AO/OTA fracture classification system, one patient had bilateral type A2 fractures, one patient had a type C2 fracture, and three patients had a unilateral type A3 fracture. None of the patients had DRUJ instability.

One patient with bilateral oblique fractures had decreased operative time and soft tissue dissection. Another patient was a victim of multiple trauma, with multiple rib fractures, pneumothorax, a maxillary fracture of the face, and thoracic aortic transection so it was important to decrease operative time after chest surgery. Severe muscle swelling was noted because of direct contusion injury in three patients.

Demographic information and radiological and clinical outcomes are shown in Table 1. The average follow-up period was 30 months (range 28–36 months). The average surgical time was 48 min (range 35–65 min). The average amount of blood loss during surgery was 27 ml (range 10–40 ml). Average time to union for each radius was 7.3 months (range 6–10 months).

**Table 1:** Demographic data of patients and their radiological and clinical outcomes

No.	Age	Sex	Side	Indication for TENs	Type of reduction	Operative time (min)	Blood loss (ml)	Duration of hospitalization (days)	Time to clinical union (month)	Supination/pronation (degree)	VAS	Quick DASH score	Follow-up period (month)
1	35	M	L	Severe soft tissue swelling	Ulna ORIF + radius CRIF	55	30	4	6	75/85	1	4.5	28
2	30	M	L	Bilateral long oblique fracture to avoid long plate and soft tissue dissection	Radius CRIF	40	10	4	6	L 75/85	1	4.5	28
			R		Radius CRIF				6	R 70/80	1	4.5	
3	43	M	L	Severe soft tissue swelling	Radius CRIF	65	30	2	8	75/80	2	4.5	30
4	64	M	L	Multiple trauma with the need to shorten operative time	Ulna ORIF + radius CRIF	35	40	26	10	45/85	3	25	36
5	43	M	R	Comminuted fracture with severe soft tissue swelling	Ulna ORIF + radius CRIF	45	25	2	8	75/80	1	4.5	28

*Abbreviations*: *VAS* visual analog scale; *Quick DASH* quick disabilities of the arm, shoulder, and hand; *ORIF* open reduction internal fixation; *CRIF* close reduction internal fixation

One patient achieved clinical union without pain or any clinical symptom at approximately 10 months postoperatively. Radiographic bone union was achieved at 18 months postoperatively, which may have resulted from the thick cortical bone and less cancellous bone as shown on the radiograph.

Immobilization with long-arm splint was performed after surgery, and the cast was removed 4 weeks later, followed by active and passive ROM of the elbow. Evaluation of functional results according to the Quick DASH scores and ROM of elbow and forearm were completed 1 year after surgery. Full ROM in flexion-extension of elbow and supination-pronation of forearm was noted in all patients expect one patient with limited ROM on forearm rotation.

The average Quick DASH score of all six injured forearms was 7.92 (range 4.5–25). The patient with the highest Quick DASH score of 25 with limited ROM was a 64-year-old male whose multiple trauma resulted in delayed rehabilitation. It was particularly difficult for him to wash his back and turn a key.

All patients were pain free and returned to their previous work approximately 4.5 months after injury except the victim of multiple trauma who retired after his accident. All patients received closed reduction, and there was no iatrogenic injury of nerves, vessels, tendons, or intraoperative fractures.

During the follow-up period, none of the patients received secondary interventions such as bone grafting or shock wave treatment. There were neither implant failures nor synostosis in our patients; however, two patients experienced skin irritation over the nail tails.

Average supination and pronation of each forearm were 69.2° (range 45°–75°) and 82.5° (range 80°–85°), respectively. According to these postoperative results, most patients successfully achieved functional recovery of the previously injured forearm, with the exception of the patient with multiple trauma, who only achieved 45° at supination of his forearm.

## Discussion

We performed CRIF in five adult patients with proximal radial shaft fractures using TENs to avoid splitting of the pronator teres muscle around the fracture site as well as to minimize PIN injury. Our results indicated that this is an alternative treatment for patients with severe muscle swelling around the fracture site after injury, as this option effectively avoids postoperative soft tissue swelling attributed to the elevation or detachment of muscles and reduces the incidence of postoperative numbness and wrist drop.

Forearm fractures are regarded as intraarticular fractures; therefore, ORIF with plate fixation remains the gold standard of treatment [1]. It is sometimes difficult to perform ORIF at certain locations of the forearm, such as the proximal radial shaft, where the pronator teres and supinator are inserted and the posterior interosseous nerve crosses.

The trauma mechanism underlying forearm fractures is often the result of direct contusion with severe soft tissue swelling. The pronator teres and supinator surrounded by the fracture site are vulnerable to transection during surgical approach. If surgeons decide to perform ORIF for proximal radial shaft fracture, the soft tissue dissection is certain to cause extensive destruction. The PIN is also at risk due to retraction instead of transection during surgery.

The Henry approach provides direct volar visualization for radial fracture reduction at the forearm supination position, with transection of pronator teres and some splitting of the common flexors. The Thomson approach may offer less destruction during soft tissue dissection when confronting the forearm fracture; however, the PIN is susceptible during this dorsal approach to the proximal radial fracture, and rotational deformity may also occur with loss of full supination of the forearm [3, 4].

Generally, TENs are popularly applied to pediatric fractures due to the thick periosteum and the increased potential for bone remodeling in children, but they are not routinely used in adults because of lack of resistance to rotational force and axial loading [7, 8]. However, TENs are appropriate for the stabilization of proximal forearm fractures, which spares the massive soft tissue dissection and avoids the possibility of PIN injury. Unlike the lower extremity with its need for weight bearing, axial loading may not remain a problem for the upper limb.

In our study, one pre-bent nail was inserted, which then became constrained by the medullary canal of the radius through 3-point pressure on the bone. The radial bowing of the radius promotes attainment of 3-point fixation on the inner aspect of the bony cortex with a single pre-contoured TEN. Because of the 3-point pressure on the bone, the pre-bent bow shape of the TEN is less likely to back out. In our cohort, the rotational force was resisted with postoperative long arm cast over 4 weeks at the forearm supination position for radial paralleling the ulna, and pre-bent TENs are designed in a hockey stick shape with the nail tip inserted into the radial head for rotational stability [15, 16].

According to the AO principles, ORIF with plate fixation is the direct reduction of a fracture site for the purpose of absolute stability and takes approximately 3 to 4 months to achieve radiographic primary bone union. Internal fixation with an intramedullary nail provides the relative stability necessary to achieve secondary bone union, and callus formation should be discovered at 3 months postoperatively during follow-up.

In our study, a small amount of callus was noted on the radiographic series approximately 3 to 4 months postoperatively, which may be attributed to the small amount of cancellous bone and thicker cortical bone content at the radio-ulnar shaft. Clinical bone union was achieved approximately 6 months postoperatively at the time of nearly painless, full range of motion, and return to previous work in our patients. In our opinion, the timing of implant removal in our patients was at least 8 months postoperatively. The patients with oblique and comminuted fractures who received CRIF with TENs demonstrated acceptable alignment of their reduction, and those patients with a transverse type fracture pattern revealed unsatisfactory anatomic reduction of the radial arch, even though there was no significant difference in functional outcomes, or supination and pronation movement between them.

The TEN is a type of non-locked intramedullary nail which provides relative stability over a fracture site. The CRIF with a non-locked intramedullary nailing technique for proximal radial fractures causes less damage to the soft tissues and less neurovascular injuries than ORIF with plating, as well as sparing the risk of re-fracture after plate removal [17]. However, the CRIF may not adequately achieve anatomic reduction, especially in radial shaft segmental fractures [16, 18, 19]. The stress sharing behavior and interfragmentary micro-motion of intramedullary implants could give rise to secondary periosteal callus formation [20].

Recently, new intramedullary radial interlocking nails for radial and ulnar fractures were designed [5]. The locked intramedullary nailing technique offers the advantages of preventing shortening and rotation in metaphyseal, comminuted, and segmental diaphyseal forearm fractures. However, CRIF with the radial interlocking nails requires screws which lock at both ends, and there is the possible risk of PIN injury during the proximal locking procedure and the risk of extensor pollicis longus and/or superficial radial nerve injuries while performing distal locking [21]. In addition, as compared with our study, the diameter of the interlocking nails is generally larger than that of the TENs, and the insertion of an interlocking nail takes more time and requires a more experienced surgical technique, concerning the preoperative planning and the intraoperative decision about the depth and width of the radial canal. Meanwhile, without a tourniquet, the medullary canal is enlarged via a reamer to allow insertion of the intramedullary interlocking nail, which is likely to cause more blood loss than a simple TEN insertion via smooth oscillating movements.

Locked intramedullary nailing is a technically more demanding procedure that involves an excessive amount of X-ray acquisition [22, 23]. In our experience, CRIF with interlocking nails usually requires more radiation exposure than with non-locked intramedullary nails in order to precisely apply the locking screws.

The efficiency of the operation for patients plays an important role. TENs application is suggested for patients with the following conditions: (1) patients with multiple trauma who require short operative time; (2) uremia patients with an ipsilateral forearm arteriovenous shunt who should avoid soft tissue dissection and extreme blood loss; (3) those with preoperative soft tissue swelling due to direct muscle contusion or strong muscularity; (4) extensive fracture of the forearm which necessitates a very long plate for ORIF; and (5) those in pursuit of cosmetic outcomes with limited soft tissue dissection. TENs can be of great benefit to the aforementioned patient populations.

### Limitations

This study had several limitations including its retrospective nature and small sample size. In addition, there was a lack of a control group of patients treated via ORIF with plate fixation and difficulties in calculating the operative time of radial fractures due to the combined ulnar fracture reduction with plate fixation in some of our cases. In addition, we could not properly quantify radiation exposures.

## Conclusions

Rigid plate fixation is still the gold standard for adult forearm fracture treatment. The use of TENs provides an alternative means for internal fixation of adult proximal radial fractures. With limited soft tissue dissection, we can avoid neuromuscular injury and produce good cosmetic outcomes. Nevertheless, the lack of absolute anatomic reduction and mechanical stability from the use of TENs necessitates 4 weeks of long-arm cast immobilization and more time for bone union. Most patients in our study achieved desirable functional outcomes, with degrees of postoperative supination and pronation that were near the normal range. A shortcoming of our study was the small sample size. Future comparative analyses of CRIF with TENs and ORIF with plates to treat adult proximal radial fractures, with large, consecutive patient cohorts, are warranted.

## Abbreviations

AO/OTA: Arbeitsgemeinschaft für Osteosynthesefragen/Orthopaedic Trauma Association
CRIF: Closed reduction and internal fixation
DRUJ: Distal radial ulnar joint
ORIF: Open reduction and internal fixation
PIN: Posterior interosseous nerve
Quick DASH: Quick Disabilities of the Arm, Shoulder, and Hand questionnaire
ROM: Range of motion
TEN: Titanium elastic nail
VAS: Visual analog scale
